# Family socioeconomic status and college attendance: A consideration of individual-level and school-level pathways

**DOI:** 10.1371/journal.pone.0284188

**Published:** 2023-04-11

**Authors:** James Tompsett, Chris Knoester

**Affiliations:** Department of Sociology, The Ohio State University, Columbus, Ohio, United States of America; University of Alabama in Huntsville, UNITED STATES

## Abstract

Inequality research has found that a college education can ameliorate intergenerational disparities in economic outcomes. Much attention has focused on how family resources impact academic achievement, though research continues to identify how mechanisms related to social class and structural contexts drive college attendance patterns. Using the Education Longitudinal Study and multilevel modeling techniques, this study uniquely highlights how extracurricular activities relate to family socioeconomic status and school contexts to influence college attendance. Altogether, sport and non-sport extracurricular participation, college expectations, and academic achievement scores, situated within unique school contexts that are driven by residential social class segregation, contribute to the cumulative advantages of children from higher SES families. The results from this study show that these cumulative advantages are positively associated with college attendance and an increased likelihood of attending a more selective school.

## Introduction

Family and other social contexts affect young adult outcomes [1–3]. For example, a family’s socioeconomic status (SES) impacts home environments, the resources available to children, the neighborhoods that they live in, and the schools that they attend [3–5]. Indeed, coming from an advantaged socioeconomic background allows for valuable privileges, opportunities, and safeguards; in contrast, coming from a disadvantaged background increases the risks of chronic stresses, strains, and hardships [2, 3, 6]. Social mobility research has shown, however, that for those who attain a bachelor’s degree, there is little association between parental resources and socioeconomic attainment [7]. Thus, in order to better understand socioeconomic inequalities, it is crucial to improve our understanding of the college attainment process—including, first of all, how and to what extent family SES links to college attendance patterns.

There are many mechanisms that lead to unequal post-secondary education opportunities that appear to be a function of SES. Compared to lower SES students, higher SES students accrue cumulative advantages from more frequently experiencing higher quality schooling, elevated academic expectations, more positive assumptions, and reinforcements of early achievements [1, 8]. Higher SES parents are also more likely to seek out and enroll their children in extracurricular activities that help prepare them for professional life and help them to stand out among their peers in recognized aspects of social and academic achievement [9–12].

Largely, these advantages stem from family situations. Yet, school contexts also independently matter; schools that primarily serve lower SES families lack funding and resources, resulting in troubling achievement gaps [5, 13, 14]. Underfunded schools are often compromised by their ability to attract and retain the most effective teachers [15, 16]. They are also less likely to be able to offer a variety of college-prep courses [17]. Relatedly, residential segregation leads to students from higher SES families concentrating in high resourced school districts [1, 13]. Thus, students from more advantaged family SES backgrounds tend to experience higher quality resources and instruction in their schools. Typically, these schooling contexts also provide superior foundations for nurturing college expectations and boosting students’ development and resumes with extracurricular experiences [18–20]. Altogether, in fact, higher SES individuals disproportionately affect and control educational systems and the establishment of meritorious criteria used to make decisions about educational advancements—and do so in ways that tend to privilege higher SES students and their families. Yet, higher SES students and their families also work very hard to use and leverage their privileges to achieve desired outcomes [21–24].

Therefore, in the present study, we apply a cumulative advantage framework to posit how family SES advantages are leveraged and then compound to affect students’ likelihood of attending college as well as their likelihood of attending more selective colleges, placing mechanisms associated with increased college attendance in context with broader SES based educational inequality. We use multilevel modeling to analyze the implications that cumulative SES advantages among a national cohort of 10^th^ graders have for: a) attending any college, and b) attending a more selective college. We consider family SES differentials, of course, but also the extent to which extracurricular activity participation levels, school-level resources, college expectations from friends and family, and students’ academic abilities matter in determining college attendance patterns. Indeed, there is evidence that these harbingers of college attendance experiences frequently flow from family SES origins [1, 3, 9, 20].

This research especially advances prior work by offering novel analyses of the associations between high school extracurricular participation and college attendance, situating this investigation within a comprehensively theorized framework, and then analyzing the extent to which family SES offers cumulative advantages to students into their high school years, in ways that are likely to affect college attendance patterns. We offer unique considerations of the mechanisms through which family SES inequalities are expected to shape college attendance patterns and do so in an advanced way with the use of multilevel modeling techniques. Related to this, we advance these inquiries by focusing on predicting both: a) any college attendance and b) the likelihood of attending a more selective college. Furthermore, we assess differences in college attendance patterns based on both sport and non-sport extracurricular activities—providing unique insight regarding a mechanism that has grounding in concerns about SES inequalities, youth development, parenting practices, and college admissions [9, 12, 25–27]. Related to this, we investigate whether participating in multiple activities provides additional benefits for college attendance patterns compared to specializing in a single sport or non-sport extracurricular activity. These advances in research are especially valuable because of the evidence of the extent to which college attendance patterns, including obtaining any college education and obtaining a more selective college education, shape later life outcomes—including adults’ socioeconomic statuses [7, 26].

## Background

The enrollment rate in college for 18- to 24-year-olds has increased by over 15% percent since 1970 [28], forcing higher education to accommodate a much larger post-secondary school population through expanding enrollments, adding campus branches, and creating entirely new higher educational institutions. Although there was some hope that the expansion of higher education could ameliorate socioeconomic inequalities, this does not seem to have occurred and inequality has continued to increase in the U.S. [3, 26, 29]. In fact, college attendance rates have increased more among higher-income families than among lower-income families [25, 26]. Also, the most prestigious schools remain disproportionately populated by the American elite despite the expanding pool of applicants [23, 26]. The selectivity of a school is important in determining long term labor market outcomes [26, 28, 30, 31]. Thus, in order to understand American inequalities, we must further examine the extent and means through which family SES leads to students’ likelihoods of attending college and attending more selective colleges.

## Theoretical framework

### Cumulative advantage

Cumulative advantage is a framework for understanding inequalities that was first presented by Merton [32] to explain how initial advantages compound over time, resulting in increasingly larger disparities in future outcomes. Scholars have applied cumulative advantage concepts to a number of realms of inequality, positing that starting with an advantage will often lead to further advantages over time—including in educational achievement [1].

Research on education has a long tradition of inquiring about how differences in family and other resources are linked to achievement inequalities. Beginning with the Equality of Educational Opportunity Report, James Coleman and his colleagues [33] noted that family resources were major predictors of educational achievement and even exerted a larger effect than school resources, it appeared. Subsequent research has also demonstrated the importance of family resources for academic achievement [22, 23, 34, 35]. Beyond individual-level family dynamics, having relatively high family SES enables parents to be more selective about the school environments that they send their children into [36, 37]. Relatedly, there is evidence that school contexts are influential in shaping patterns of college attendance [26, 29, 31, 38].

In the present study, cumulative advantage considerations focus on how family SES leads to different pathways in educational experiences and influences a student’s likelihood of attending any college, a four-year versus a two-year college, and a more selective college. We highlight the relevance of: a) school contexts, b) extracurricular involvement, c) academic expectations, and d) academic achievement—although we also discuss other basic ways through which family SES may lead to different patterns of college attendance.

### School contexts and college attendance

One primary pathway through which family SES operates in shaping students’ college attendance patterns is through placing students within wealthier or poorer schools, that often offer different environments for enriching students’ experiences [24, 36–38]. While decreases in funding have disproportionately forced lower resourced schools to cut all but the necessities out of their curricular and extracurricular programming, schools serving more advantaged SES populations have continued to offer a wide variety of curricular and extracurricular activities that aid student development. Relatedly, schools in lower SES districts are more likely to struggle to maintain their facilities, allocating their modest resources to keeping facilities open rather than being able to incorporate new technology or academic resources into their educational offerings [5, 34, 39, 40]. Teacher quality and continuity is another factor that impacts the schooling that can be offered at low SES schools. The most qualified teachers are able to avoid working in low SES schools, which are considered to be high stress environments, while teachers transfer from these institutions at higher rates than from schools serving high SES students [15, 41]. Teacher turnover hinders the quality of education that students receive, and a lack of continuity particularly impacts poorer students [42].

There are also school level differences that offer cumulative advantages due to disparities in educational climates, discipline issues, social capital, role modeling, and socialization messages—although our analyses in this study only capture structural characteristics at the school level [31, 43]. Thus, residential patterns grouping well-resourced families together further enhance the educational opportunities of high SES students who disproportionately attend high resourced schools [44]. Brian Levy and his colleagues [45] suggest that enhanced collective efficacy among higher SES community members lead to higher levels of college graduation due to the shared values and expectations of attaining a college degree. In order for college expectations to consistently translate into college attendance, students may need to be in school environments with many college going peers [46]. In addition, high expectations from teachers positively affect achievements in the classroom, while low expectations can negatively impact educational achievements [47]. Students’ social class statuses seems to shape teachers’ expectations, regardless of other salient academic factors [48].

### Educational expectations, educational achievements, and college attendance

Family SES also matters in more direct ways at the individual level as it may influence the educational expectations that surround students, students’ own educational achievements, and their participation in extracurricular activities. Although these mechanisms are expected to translate into different college attendance patterns along SES lines, individuals in the U.S. largely view these processes as meritocratic, instead of unequal [26]. That is, public education, widely available extracurricular activities, and expanded access to college are thought to provide equitable opportunities for all to obtain a good foundational education through high school, achieve necessary and desired credentials for college admittance, and attend an appropriate college, if one wants and so chooses, on the pathway to realizing deserved labor market outcomes. Yet, ever emerging evidence suggests that those from advantaged backgrounds are primed, focused, intentional, and able to leverage their knowledge, privileges, and resources in order to disproportionately take advantage of educational and extracurricular systems and opportunities that consequently results in superior educational outcomes for higher SES students—with administrators, power brokers, and other guardians of the systems none too upset or resistant to these dynamics [21–24, 49].

First, educational expectations—from parents, students, and significant others—impact the achievements and aspirations of students [43, 50]. Expectations matter because students with higher educational expectations are more likely to attend college and expectations for college attendance seem to differ by family SES in ways that advantage more privileged individuals [50–52]. Nevertheless, students who recognize the educational path of a parent(s) who obtained higher education will generally internalize assumptions about college expectations, indirectly [53].

Not only are parents with higher SES more likely to expect their children to attend college, but they also are more likely to confer tangible benefits and resources to them that enhance educational expectations and achievements. These include educational resources (e.g., books, computers), experiences (e.g., learning opportunities), and interactions (e.g., conversations). For example, higher educated parents are more likely to read to their child at a young age, which encourages reading and translates to increased performance in school [54, 55]. Also, family SES can affect the number of books that parents can provide in the home, which has been shown to impact reading abilities as well [56–58]. Exposure to educational technology also varies by SES, with advantaged students more likely to have access to technology and become comfortable with using it to achieve school goals [8].

In addition to these more explicitly educational advantages, there are also more indirect effects of family SES on learning environments. Children from lower SES families may be more likely to have their educational focus and achievements compromised by devastating stressors, such as having to overcome health related detriments to their educational achievement [2, 59, 60]. Hunger and poor health harm students’ ability to focus during class, and poor health is likely to prevent students from attending school in the first place. Housing precarity is also linked to low SES status, which also leads to decreased attendance, and inherently diminishes educational continuity linked to higher achievement [5]. Lower SES families are also more likely to suffer from stressors associated with living in low-income neighborhoods, such as pollution, crime, and disorder [2, 61]. The difficulties prevalent among low SES families elevates role strains for parents and often impedes their ability to provide high levels of support for their child’s academic achievement [2, 56]. In sum, advantaged families do not typically have to navigate as many extreme challenges that impact lower SES students and instead can more fully focus on nurturing preferred college attendance patterns.

Altogether, these experiences are anticipated to lead to cumulative (dis)advantages that systematically affect educational achievements. For example, Feldman and Matjasko [62] note that family SES is highly correlated with grade point averages. In addition, standardized test scores, the other main achievement measure used to assess college applicants, are so highly linked to family SES that some scholars view SAT and ACT tests as products and more precisely vessels for justifying class inequalities [26, 30, 63, 64]. The present study uniquely highlights the roles of extracurricular activities in the U.S. and how students from advantaged SES backgrounds may leverage their corresponding knowledge bases, resources, and abilities into disparities in post-secondary enrollment [9, 11, 65].

### Extracurricular activities and college attendance

Indeed, higher SES parents appear to be increasingly investing in and encouraging extracurricular activities as important pathways to college attendance and into more selective school attendance [9, 11, 31, 65]. Of course, these investments are enabled by SES-related resources [9, 12, 31]. Participating in extracurricular activities is associated with several positive outcomes, including increased grade point averages [66–68], lower dropout rates [69–71] and some evidence of higher levels of college attendance and selective college attendance [29, 49, 72].

Recent research has remained attentive to the association between extracurricular participation and academic outcomes, providing useful insight to the educational benefits of participating in extracurricular activities; notably, however, there is a lack synthesis for how different types of activities, and participating in multiple activities, drive associations with college attendance. For example, Shrifrer and colleagues [73] found that there were persistent college attending benefits for students who participated in high school sports from the 1980s into the 2000s, and gaps in participation by SES increased over time, suggesting that this mechanism for educational inequality is becoming stronger. Gibbs and colleagues [74] more thoroughly accounted for a variety of extracurricular offerings in their analysis, emphasizing how peer networks associated with extracurricular participation drive differences in academic achievement. Their findings suggest that specifically academic activities contribute to higher GPAs and increased college attendance. Importantly, studies have begun to differentiate between sport and non-sport activities when assessing social and academic benefits [25, 64, 75, 76]. Yet, only Fredricks and Eccles [77] consider how multiple forms of extracurricular participation relate to college attendance. This study used data from Maryland in the 1990s with a sample of close to one thousand to find that multiple forms of participation are positively associated with college attendance. We seek to expand their discussion by exploring whether these associations hold in a larger, national dataset and whether participation influences the selectivity of colleges attended.

Although there is equivocal evidence about the extent to which extracurricular activities, and different numbers and types of activities, may affect college attendance patterns per se, there is good reason to think that they may matter substantially. Participating in extracurricular activities can aid student development in ways that can improve academic aspirations, commitments, and performance. In response to increased competition in college admissions, higher SES families have adapted by seeking to improve their children’s credentials [23]. Kaisa Snellman and colleagues [11] explicitly connect the increased disparities in extracurricular participation levels between higher and lower SES students to the inequalities in college attendance by family SES. There is some evidence that disadvantaged students who are able to participate in extracurricular activities normally unavailable to low-income students seem to significantly increase their likelihood of attending college, at least [78]. Further, these suggestions of a family SES, extracurricular participation, and college attendance pattern relationship are consistent with evidence that college admissions officers actively seek out potential applicants who not only participate in extracurricular activities, but also signal by their activity participation that they are from higher SES backgrounds [19, 29].

### Other SES links to college attendance

Beyond the specific mechanisms discussed above, there are a number of other pathways through which family SES is expected to lead to different college attendance patterns. Although our analyses cannot tease out evidence of these particular pathways, we introduce them as potential explanations for residual associations between family SES and college attendance patterns, after we focus our analysis on the aforementioned processes. At the most basic level, family SES determines the extent and likelihood that a child can afford to go to college and to a more selective college. The costs of college attendance have skyrocketed, and the numerous fees involved in test taking, applying, and visiting schools are substantial and increasing, as well [26, 29, 31, 79]. Because of their increased likelihood of being able to pay more for tuition and to offer donations, wealthier families are also more likely to be recruited to attend college and more selective colleges. Higher SES families are also more likely to benefit from legacy admissions, early admissions, and familiarity with the processes of applying for college [26, 29, 31]. Higher levels of family SES are also associated with various forms of social and cultural capital that enables individuals from more privileged families to benefit from their social connections and patterns of social interactions [9, 26]. While a focus of this study is to interrogate different forms and combinations of extracurricular participation, we recognize it is only one potential confounder in the link between family SES and college attendance. Thus, we anticipate that family SES is likely to be positively correlated with the likelihood of attending college, and a more selective college, even after accounting for differences in school contexts, college expectations, academic abilities, and extracurricular activities.

### Hypotheses

Overall, our conceptual framework and previous research lead to us emphasizing the following hypotheses:

H1: SES will be positively associated with the likelihood of attending college and of attending a more selective college, both at the individual level and the school level.
H2: Both sport and non-sport extracurricular activities will be positively associated with the likelihood of attending college and of attending a more selective college. Still, relationships between family SES and patterns of college attendance will persist.
H3: College expectations and academic abilities will be positively associated with the likelihood of attending college and of attending a more selective college. Still, relationships between family SES and patterns of college attendance will persist.

## Data and methods

### Data

To address our research questions, we use the Education Longitudinal Survey (ELS), a nationally representative data set that first sampled 752 out of an initially identified 1,221 US high schools [80, 81]. From the school sample, the ELS then selected a student sample, collecting information on approximately twenty-six sophomores from each school. After eliminating cases with missing data on key variables, the main sample for this study yields 10320 student respondents within 682 schools; 67 schools reported missing values for their socioeconomic composition, and three schools no longer have cases after removing missing data. The student responses are matched with parent and school administrator responses. For analyses that require information about the type of college that students attended, our sample size is smaller (i.e., *n* = 7,118) because of a lack of information about the characteristics of some colleges.

The first wave of data was collected in 2002, when students were in 10^th^ grade. The ELS also collected follow-up data in 2004 and 2006. We draw upon these longitudinal data to consider how the cumulative advantages of family SES through the 10^th^ grade years of students influence their college attendance patterns, as reported in 2006. The ELS data, despite their age, are particularly useful for the purposes of our study because they offer nationally representative information about 10^th^ graders’ family SES statuses, school contexts, college expectations, academic abilities, and extracurricular participation levels—as well as students’ eventual college attendance patterns. Thus, they are uniquely appropriate for our analyses.

#### Variables

All variables are created from 2002 reports except for our 12^th^ grade extracurricular variables, taken in 2004, and our dependent variables, which were created from 2006 reports.

There are three dependent variables for our analyses: a) reports of any college attendance, b) reports of initially attending a two-year or a four-year college—measured two years after students’ 12^th^ grade years, and c) the selectivity of the first postsecondary institution attended. *College attendance* indicates whether respondents reported that they have ever attended a postsecondary school (1 = *yes*). *Type of college attendance* distinguishes the type of their first postsecondary institution, with responses that include options for institutions that offer educations of four or more years, at least two but less than four, and less than two years. Reponses for less than two years are coded as not attending college for our analysis. Mutually exclusive dummy variables indicate type of college attended: a) no college, b) a two year college, or c) a four year college. *College selectivity* further specifies the type of college attended, using student transcript data to identify the first postsecondary institution they attended. This dependent variable uses the Carnegie selectivity measurement. “Highly selective” schools represent institutions in the top fifth for first year student test scores; “moderately selective” schools represent institutions in the second and third fifths of first year student test scores; “inclusive” schools accept students from a wide range of academic preparation and achievement. This dependent variable is constructed as an ordinal measures with the following categories: two year college attendance, inclusive four year college attendance, moderately selective four year college attendance, and highly selective four year college attendance.

The primary independent variables of interest in our analysis involve *family SES* and participation in extracurricular activities. We use a composite scale for SES provided by the ELS that is based on father’s education, mother’s education, family income, father’s occupation, and mother’s occupation when the student was in 10^th^ grade. The component indicators are weighted equally in the construction of the SES measure and missing component indicators are imputed. Extracurricular participation variables measure whether students played a sport in 10^th^ grade (1 = *yes*), or in 12^th^ grade (1 = *yes*), and whether they participated in a non-sport extracurricular activity in 10^th^ grade (1 = *yes*) or in 12^th^ grade (1 = *yes*). The non-sport participation variable is constructed from survey responses about participation in music, drama, student government, or the school paper. We also include dichotomous indicators that represent if students participated in multiple sports or multiple non-sport extracurricular activities.

Other primary predictors for this study include measures of school level SES, college expectations from the students and their significant others, and students’ standardized test scores. For *school level SES*, we use the percent of the student body that is on free lunch, as reported by school administrators. These responses form an ordinal variable (1 = *0–20% on free lunch*; 4 = *>75% on free lunch*). Measures of *college expectations* include a self-report from students (1 = *yes*) as well as a constructed count variable that indicates the number of significant others from among parents, friends, guidance counselors, and coaches who think that the student should attend college. There is also an indicator of students’ academic abilities from *standardized test scores*. This measure is constructed by taking the average of reading and math standardized test scores in the 9^th^ grade, re-standardized to a national average of 50 and a standard deviation of 10.

We also include a number of control variables in our analytic models. These include indicators of gender (1 = *female*), race/ethnicity (coded as mutually exclusive categories for *white*, *non-Latinx*, *black*, *Asian American*, *Latinx*, *or other race/ethnicity*; white is used as the reference category), and parental marital status (coded as *single/cohabiting/married*; married is used as the reference category). We also control for the presence (1 = *yes*) of a *cognitive disability*, *physical disability*, and language barrier (i.e. *English as a second language*).

At the school level, we control for public/private school categorization, region and urbanicity. We distinguish attendance at a *private school* (public school used as reference) and at an *urban* or *rural* school, as opposed to a suburban school (used as reference category). Finally, we consider the region of the school and denote whether it is in the Midwest, the West, or the South (Northeast used as reference).

## Results

We begin our analysis by predicting college attendance patterns in three stages, each of which considers both school level and individual level predictors. First, we use multilevel binary logistic regressions to predict any college attendance in a series of nested models. Then, we present results from multilevel binary logistic regressions that predict the likelihood of attending a four-year versus a two-year college, among those students who attended college, in a series of nested models. In sensitivity analyses, we also used multilevel multinomial logistic regressions (results not shown) to compare the likelihoods of a) not attending college, b) attending a two-year college, and c) attending a four-year college. For the sake of clarity and parsimony, we do not present those results, however—they reinforce the findings that are presented. Last, we predict college students’ likelihood of enrolling in a more selective school with multilevel ordinal logistic regressions. Finally, we conclude our presentation of results by displaying the full descriptive statistics for our sample and offer a more straightforward summary of their apparent relevance for college attendance patterns.

### Socioeconomic status and any college attendance

We predict the likelihood of 10^th^ grade students attending any college within the next four years with a focus on how family SES, and other factors that are thought to be extensions of family SES, matter. Table 1 displays the results of logistic regressions that predict these likelihoods in a series of nested models that: a) first offer predictions with individual and spatial background characteristics, b) then add family SES as the primary independent variable, c) next add school level predictors, with a focus on school level SES, and d) finally add indicators of college expectations, test scores, and extracurricular activities.

**Table 1:** Multilevel logistic regressions predicting the likelihood of attending college.

	Model 1			Model 2			Model 3			Model 4		
	b	SE	OR	b	SE	OR	b	SE	OR	b	SE	OR
*School level*												
Low SES composition							-.12**	(.05)	.89	-.07	(.04)	.93
Private							1.06***	(.12)	2.89	.78***	(.11)	2.18
Urban^1^	.25**	(.09)	1.28	.29*	(.08)	1.21	.04	(.09)	1.04	.05	(.08)	1.05
Rural^1^	-.37***	(.11)	.69	-.16	(.09)	.85	-.04	(.09)	.96	-.09	(.09)	.92
Midwest^2^	-.25	(.13)	.78	-.12	(.11)	.89	-.08	(.11)	.92	.03	(.10)	1.03
South^2^	-.30*	(.12)	.74	-.25*	(.10)	.78	-.14	(.11)	.87	-.03	(.10)	.97
West^2^	-0.23	(.14)	.79	-.17	(.12)	.84	-0.05	(.11)	.95	.09	(.11)	1.09
*Individual level*												
Female^3^	.40***	(.05)	1.50	.49***	(.05)	1.64	.50***	(.06)	1.67	.41***	(.06)	1.50
Black^4^	-.40***	(.09)	.67	-.08	(.09)	.92	.07	(.11)	1.07	.31**	(.10)	1.36
Latinx^4^	-.74***	(.09)	.47	-.35*	(.10)	.70	-.26**	(.09)	.77	.00	(.10)	1.00
Asian^4^	.44***	(.13)	1.55	.63***	(.14)	1.88	.75***	(.14)	2.13	.70***	(.14)	2.02
Other ethnicities^4^	-.62***	(.11)	.55	-.53***	(.11)	.59	-.49***	(.12)	.61	-.46***	(.14)	.63
Cohabit^5^	-.74***	(.07)	.47	-.63***	(.07)	.53	-.58***	(.08)	.56	-.48***	(.08)	.62
Single^5^	-.57***	(.06)	.56	-.30***	(.07)	.74	-.38***	(.08)	.76	-.16**	(.07)	.85
Cognitive Disability	.92***	(.09)	.40	-.94***	(.10)	.39	-.94***	(.11)	.39	-.23*	(.11)	.79
Physical Disability	-.31	(.17)	.73	-.41	(.17)	.66	-.38	(.20)	.68	-.17	(.18)	.84
ESL	-.31*	(.09)	.74	.09	(.10)	1.09	.13	(.11)	1.14	.32**	(.10)	1.47
SES				1.23***	(.05)	3.40	1.13***	(.05)	3.10	.71***	(.05)	2.04
College Expectations										.78***	(.06)	2.17
Other’s Expectations										.08***	(.01)	1.08
Test scores										.07***	(.00)	1.07
10th grade sport										.21**	(.08)	1.23
10th grade non-sport										.07	(.08)	1.07
12th grade sport										.60***	(.07)	1.82
12th grade non-sport										.42***	(.07)	1.51
Multisport										.03	(.09)	1.03
Multi non-sport										.01	(.12)	1.01
Variance component		0.63			0.31			0.21			0.14	

*Note*: N = 10,320. P < .05

**p < .01

***p < .001. Reference categories: 1-Suburban, 2-Northeast 3-Male, 4-White, 5-Married.

Model 1 of Table 1 offers baseline estimates of the associations between background characteristics and college attendance patterns. It reveals evidence that the odds for females (OR = 1.50, *p* < .001) attending college are 50% higher than those for males. Also, racial/ethnic minority students, with the exception of Asian Americans (OR = 1.55, *p* < .001), are less likely to attend college than white students. Compared to students with married parents, students who live with a single parent (OR = .56, *p* < .001) or with a parent who is cohabiting (OR = .47, *p* < .001) are less likely to attend college. There is also evidence that having a cognitive disability (OR = .40, *p* < .001) or a language barrier (OR = .74, *p* < .05) is negatively associated with college attendance, as anticipated. Considering school contexts, compared to attending a suburban school, students in urban schools are more likely to attend college (OR = 1.28, *p* < .01), while students in rural schools are less likely to attend college (OR = .69, *p* < .001). Students in the South (OR = .74, *p* < .05) are less likely to attend college than students in the Northeast.

In Model 2, we introduce a primary independent variable, family SES. As anticipated by our first hypothesis, SES is positively associated with college attendance (OR = 3.40, *p* < .001). In particular, a one unit increase in family SES is associated with having odds for the likelihood of attending college that are 3.4 times as great as the odds for students with family SES that is one unit lower on the scale.

In Model 3 of Table 1, key school level characteristics are added to the multilevel model. Our focus here is on the school level indicator of socioeconomic status, which is associated with college attendance, providing further support for our first hypothesis. That is, the percentage of students on free lunch is negatively associated with an individual student’s likelihood of attending college (OR = 0.89, *p* < .01). Notably, measures of region and urbanicity are no longer significant after accounting for school SES and private status, suggesting that SES may have been driving the region and urbanicity findings.

In Model 4 of Table 1, indicators of extracurricular activities, college expectations, and test scores are added into the predictive model. We find initial evidence in support of our second hypothesis. Participating in a sport in 10^th^ grade (OR = 1.23, *p* < .01) and 12^th^ grade (OR = 1.82 *p* < .001) are each positively associated with college attendance. Participating in a non-sport extracurricular activity in 12^th^ grade is positively associated with college attendance (OR = 1.51, *p* < .001), though this is not the case for 10^th^ grade participation Participating in multiple sports or multiple non-sport activities is not further associated with college attendance.

Model 4 of Table 1 also demonstrates, consistent with our third hypothesis, that student’s college expectations (OR = 2.17, *p* < .001), others’ college expectations for the student (OR = 1.08 *p* < .001), and test scores (OR = 1.06, *p* <. 001) are positively associated with college attendance. In fact, after the addition of these extracurricular, college expectations, and academic ability factors into the analytic model, family SES appears to be less predictive of the likelihood of college attendance, itself (i.e., the log odds for the family SES coefficient decrease from 1.13, *p* < .001 to .71, *p* < .001). Still, it remains relevant—as anticipated.

### Socioeconomic status and attending a four-year versus a two-year college

Next, we turn to predicting the likelihood of attending a four-year versus a two-year college. Table 2 displays the results from the corresponding logistic regression results that are comparable to those that are presented in Table 1 but focus on predicting the relative likelihood of attending a four-year versus a two-year college among students who went to college.

**Table 2:** Multilevel logistic regressions predicting the likelihood of attending a four-year versus a two-year college among college attendees.

	Model 1			Model 2			Model 3			Model 4		
	b	SE	OR	b	SE	OR	b	SE	OR	b	SE	OR
*School level*												
Low SES composition							-.15**	(.05)	.86	-.09	(.06)	.91
Private							.47***	(.11)	1.59	.32**	(.11)	1.39
Urban^1^	.57***	(.10)	1.77	.55***	(.09)	1.73	.47***	(.09)	1.60	.58***	(.19)	1.78
Rural^1^	-.30*	(.12)	.74	-.11	(.11)	.86	-.08	(.11)	.92	-.15	(.12)	.86
Midwest^2^	-.34*	(.13)	.71	-.28*	(.13)	.76	-.26	(.12)	.77	-.20	(.13)	.82
South^2^	-.35**	(.13)	.70	-.36**	(.12)	.70	-.28	(.12)	.76	-.18	(.12)	.83
West^2^	-.77***	(.14)	.46	-.74***	(.14)	.48	-.66***	(.13)	.52	-.64**	(.14)	.53
*Individual level*												
Female^3^	.09	(.05)	1.09	.15**	(.06)	1.16	.16**	(.05)	1.17	.17**	(.06)	1.18
Black^4^	-.16	(.10)	.85	.06	(.10)	1.06	.15	(.10)	1.17	.75***	(.11)	2.13
Latinx^4^	-.64***	(.10)	.53	-.41***	(.11)	.67	-.36***	(.11)	.70	-.05	(.11)	.95
Asian^4^	.28*	(.12)	1.32	.34*	(.12)	1.41	.40**	(.12)	1.49	.49**	(.13)	1.64
Other ethnicities^4^	-0.08	(.13)	.92	-.04	(.13)	.96	-.02	(.13)	.98	.11	(.14)	1.11
Cohabit^5^	-.56***	(.08)	.57	-.43***	(.08)	.65	-.41***	(.08)	.67	-.31***	(.09)	.74
Single^5^	-.34***	(.07)	.71	-.15	(.07)	.86	-.14	(.07)	.87	-.05	(.08)	.95
Cognitive Disability	-1.18***	(.12)	.31	-1.24***	(.12)	.29	-1.25***	(.13)	.29	-.54***	(.14)	.58
Physical Disability	-1.00***	(.22)	.37	-1.14***	(.23)	.32	-1.14***	(.23)	.32	-.85***	(.25)	.43
ESL	-.40**	(.10)	.67	-.17	(.10)	.84	-.13	(.10)	.88	.06	(.11)	1.06
SES				.80***	(.04)	2.23	.71***	(.04)	2.03	.41***	(.05)	1.50
College Expectations										.52***	(.08)	1.67
Other’s Expectations										.05***	(.01)	1.05
Test scores										.10***	(.00)	1.10
10th grade sport										.25**	(.08)	1.28
10th grade non-sport										.05	(.08)	1.10
12th grade sport										.44***	(.08)	1.55
12th grade non-sport										.31***	(.07)	1.37
Multisport										.04	(.09)	1.04
Multi non-sport										.09	(.10)	1.10
Variance component		0.75			0.59			0.54			.48	

Note: N = 7984

*p < .05

**p < .01

***p < .001. Reference categories: 1-Suburban, 2-Northeast 3-Male, 4-White, 5-Married.

Model 1 of Table 2 reveals similar findings involving the associations between background characteristics and attending a four-year college versus a two-year college, compared to the findings uncovered when predicting any college attendance. That is, there is evidence that Latinx students are less likely (OR = .53, *p* < .001) than white students to attend a four-year college as opposed to a two-year college and Asian American students are more likely (OR = 1.32, *p* < .05) than white students to attend a four-year college as opposed to a two-year college. Compared to students with married parents, students who live with a single parent (OR = .71, *p* < .001) or with a cohabiting parent (OR = .57, *p* < .001) are less likely to attend four-year versus a two-year college. There is also evidence that having a cognitive disability (OR = .31, *p* < .001), a physical disability (OR = .37, *p* < .001) or a language barrier (OR = .67, *p* < .01) is negatively associated with college attendance at a four-year school versus a two-year school.

Once again, in Model 2, we introduce our first primary independent variable, family SES. As predicted in our first hypothesis, family SES is positively associated with college attendance at a more selective school (OR = 2.23, *p* < .001). A one unit increase in family SES leads to more than a doubling of the odds of attending a four-year versus a two-year college.

In Model 3 of Table 2, key school level measures of socioeconomic status are added to the multilevel logistic regression model. School level socioeconomic statuses are positively associated with the likelihood that a student attends a four-year as opposed to a two-year college, providing further support for our first hypothesis. The likelihood of attending a four-year college seems to decrease as the percentage of students at one’s school that are on free lunch increases (OR = 0.86, *p* < .01).

In Model 4 of Table 2, indicators for extracurricular activities, college expectations and test scores are added to the equation. As predicted by our second hypothesis, there is evidence that participating in school sports in 10^th^ grade (OR = 1.28, *p* < .01) and 12^th^ grade (OR = 1.55, *p* < .001) as well as non-sport extracurricular activities in 12^th^ grade (OR = 1.55, *p* < .001) are positively associated with college attendance at a four-year as opposed to a two-year school. Student’s college expectations (OR = 1.67, *p* < .001), others’ college expectations for the student (OR = 1.05, *p* < .001), and test scores (OR = 1.10, *p* < .001) are also positively associated with college attendance at a four-year as opposed to a two-year school, consistent with our third hypothesis. Furthermore, the consideration of these factors reduces the size of the coefficient for family SES in predicting the likelihood of college attendance at a four-year versus a two-year school (i.e., the log odds for the family SES coefficient decrease from .71, *p* < .001 to .41, *p* < .001)—although family SES remains significant, as expected.

Finally, we predict the selectivity of college attended, among college students, with multilevel ordinal logistic regressions. First, Model 1 of Table 3 reveals findings that are very consistent with the previously reported patterns of educational inequalities. Compared to white students, black and Latinx students are less likely to attend more selective colleges (OR = .62, *p* < .001; OR = .51, *p* < .001, respectively), while Asian American students are more likely to attend more selective colleges (OR = 1.47, *p* < .001). Also, living with a single parent (OR = .72, *p* < .001) or with a parent who is cohabiting (OR = .60, *p* < .001), compared to being married, is negatively associated with college selectivity. In addition, having a cognitive disability (OR = .30, *p* < .001) or a physical disability (OR = .31, *p* < .001) is negatively associated with attending a more selective college. Attending an urban high school is positively associated with college selectivity (OR = 1.63, *p* < .001), while attending a rural high school is negatively associated with college selectivity (OR = .75, *p* < .05), compared to attending a suburban high school. Attending a high school in the in the Midwest (OR = .70, *p* < .01), South (OR = .75, *p* < .05), and West (OR = .47, *p* < .001) is negatively associated with college selectivity, compared to attending a school in the Northeast.

**Table 3:** Multilevel ordinal logistic regressions predicting college students’ likelihoods of enrolling at a more selective school.

	Model 1			Model 2			Model 3			Model 4		
	b	SE	OR	b	SE	OR	b	SE	OR	b	SE	OR
*School level*												
Low SES composition							-.24***	(.05)	.77	-.15**	(.05)	.86
Private							.48***	(.10)	1.62	.40***	(.10)	1.49
Urban^1^	.49***	(.10)	1.63	.46***	(.09)	1.58	.39***	(.09)	1.47	.48***	(.09)	1.60
Rural^1^	-.29*	(.12)	.75	-.14	(.11)	.87	-.06	(.11)	.94	-0.14	(.10)	.87
Midwest^2^	-.35***	(.13)	.70	-.29*	(.12)	.75	-.26*	(.11)	.77	-.24*	(.11)	.79
South^2^	-.29*	(.13)	.75	-.31**	(.11)	.74	-.20	(.13)	.82	-.13	(.13)	.87
West^2^	-.75***	(.15)	.47	-.70***	(.12)	.50	-.60***	(.13)	.55	-.61***	(.13)	.55
*Individual level*												
Female^3^	.05	(.05)	1.05	.10*	(.05)	1.10	.11*	(.05)	1.11	.13*	(.05)	1.14
Black^4^	-.48***	(.09)	.62	-.29**	(.09)	.75	-.19	(.09)	.83	.36***	(.10)	1.43
Latinx^4^	-.67***	(.10)	.51	-.47***	(.10)	.62	-.43***	(.10)	.65	-.11	(.10)	.90
Asian^4^	.39***	(.12)	1.47	.43***	(.11)	1.53	.49***	(.11)	1.63	.57***	(.11)	1.77
Other ethnicities^4^	-.11	(.12)	.89	-.09	(.12)	.92	-.06	(.12)	.94	.06	(.12)	1.06
Cohabit^5^	-.51***	(.07)	.60	-.42***	(.07)	.69	-.35***	(.07)	.71	-.25***	(.07)	.78
Single^5^	-.33***	(.06)	.72	-.14*	(.06)	.87	-.13*	(.06)	.88	-.02	(.07)	.98
Cognitive Disability	-1.20***	(.12)	.30	-1.26***	(.13)	.28	-1.26***	(.13)	.28	-.54***	(.13)	.58
Physical Disability	-1.18***	(.23)	.31	-1.31***	(.23)	.27	-1.31***	(.23)	.27	-1.07***	(.23)	.35
ESL	-.26	(.09)	.77	-.02	(.09)	.98	.01	(.10)	1.01	.21*	(.10)	1.22
SES				.81***	(.04)	2.24	.76***	(.04)	2.12	.44***	(.04)	1.53
College Expectations										.49***	(.08)	1.63
Other’s Expectations										.04***	(.01)	1.04
Test scores										.11***	(.00)	1.11
10th grade sport										.23***	(.07)	1.26
10th grade non-sport										.09	(.06)	1.12
12th grade sport										.41***	(.06)	1.51
12th grade non-sport										.25***	(.06)	1.28
Multisport										-.04	(.06)	.96
Multi non-sport										.12	(.08)	1.13
Variance component		0.9			0.65			0.57			0.55	

*Note*: N = 7118. p < .05

**p < .01

***p < .001. Reference categories: 1-Suburban, 2-Northeast 3-Male, 4-White, 5-Married.

In Model 2 of Table 3, we introduce our first primary independent variable, family SES. Consistent with Hypothesis 1, family SES is positively associated with college selectivity (OR = 2.24, *p* < .001).

In Model 3 of Table 3, school level SES and private/public status are added to the equation. Results provide further evidence in support of our first hypothesis, demonstrating that high school SES is positively associated with attending a more selective college. For example, the percentage of students on free or reduced lunch (OR = .77, *p* < .001) is negatively associated with college selectivity. Also, attending a private high school (OR = 1.62, *p* < .001) is positively associated with college selectivity.

Model 4 introduces more individual level covariates expected to be associated with attending a more selective college, including key measures of extracurricular participation. Evidence that sports participation in 10^th^ (OR = 1.26, *p* < .001) and 12^th^ grade (OR = 1.51, *p* < .001) is positively associated with attending a more selective college supports our second hypothesis. Additionally, participating in non-sport extracurricular activities in 12^th^ grade (OR = 1.28, *p* < .001) is positively associated with attending a more selective college. There is no evidence that playing multiple sports or participating in multiple non-sport extracurricular activities increases the likelihood of attending a more selective college. A student’s college expectations (OR = 1.63, *p* < .001), college expectations for the student from others (OR = 1.04, *p* < .001) and test scores (OR = 1.11, *p* < .001) are positively associated with attending a higher category of college selectivity. As predicted by our third hypothesis, family SES (OR = 1.53, *p* < .001) remains significantly associated with attending a college in a higher category of selectivity, but the coefficient is reduced after accounting for key school level and individual level covariates, as expected (i.e., the log odds for the SES coefficient decrease from .81, p < .001 to .44, p < .001 across Models 2–4).

### Descriptive statistics

Finally, before proceeding to discuss our results and their implications, we provide an overview of the frequencies and averages of the variables used in our analysis. Table 4 presents these descriptive statistics. In order to highlight some of the notable differences in college attendance patterns, we also offer comparisons of the descriptive statistics for those who attended college and those who did not, accompanied by significance tests indicating when the mean/percentage of any college attendance differs from the overall sample. We display full sample characteristics in the “Full Sample” column.

**Table 4:** Descriptive statistics for all variables used in the analyses.

	Full Sample	Any College	No College	
	M(SD)/%	M(SD)/%	M(SD)/%	
*Individual Level Variables*				
(No College)	22.46%			
Any College	77.54%			
—2 Year College	27.10%			
—4 Year College	50.43%			
College Selectivity	2.43(1.21)			
(Male)	47.95%	73%	27%	***
Female	52.05%	80%	20%	***
(White)	60.10%	81.04%	18.96%	
Black	11.98%	70.79%	29.21%	***
Asian	9.22%	87.08%	12.92%	***
Latinx	13.32%	65.60%	34.40%	***
Other Ethnicities	5.38%	67.57%	32.42%	***
SES	0.11(.75)	.24(.73)	-.37(.60)	***
(Married)	62.50%	83.26%	16.74%	
Single	22.14%	69.85%	30.15%	***
Cohabit	15.36%	65.68%	34.32%	***
Cognitive Disability	6.59%	57.35%	42.64%	***
ESL	15.63%	73.71%	26.29%	***
Physical Disability	2.02%	60.58%	39.42%	***
Expect College	77.64%	85.60%	14.40%	***
Others Expect College	4.09(2.38)	4.41(2.38)	3.00(2.50)	***
Test Scores	52.10(9.73)	54.17(9.10)	44.96(8.73)	***
10th Grade Sport	56.85%	84.17%	15.83%	***
10th Grade non-sport ECA	36.82%	84.76%	15.24%	***
12th Grade Sport	39.43%	88.35%	11.65%	***
12th Grade non-sport ECA	44.49%	86.82%	13.18%	***
Multisport	29.47%	85.14%	14.18%	***
Multiple non-sport ECAs	12.19%	86.96%	13.04%	***
*School Level Variables*				
Low SES Composition	1.61(.97)	1.49(.78)	1.86(.95)	***
Private	23%	93%	7%	***
(Urban)	31%	80%	20%	
Suburb	49%	77%	23%	***
Rural	20%	71%	29%	***
(Northeast)	17%	84%	16%	
Midwest	26%	79%	21%	
South	38%	78%	22%	
West	19%	78%	22%	
N = 10,320				

*Note*: Significance levels represent mean/% differences of any college attendance based on high school characteristics

*p < .05

**p < .01

****p < .001.

Notably,77% of the 10^th^ grade cohort reported attending college. Specifically, 23% of students did not attend college, 29% attended a two-year college, and 48% attended a four-year college. Also, correlations involving our main predictors of interest are apparent. For instance, compared to those who did not attend college, college attendees were from more privileged SES backgrounds (i.e., .24 vs. -.37) and attended high schools with lower proportions of low SES students (i.e., 1.49 vs. 1.86). In addition, 88% of students who participated in 12^th^ grade sports attended college, compared to 77.5% of the overall sample. This pattern is clear for non-sport extracurricular activities as well, where over 86% of students who participated in non-sport activities attended college. Finally, in line with our results, college attendees had higher test scores (54.79 vs. 44.96) and expectations from others of their going to college (4.41 vs. 3.00), compared to those who did not go to college. College attendees were also disproportionately likely to expect to go to college, early in their high school careers (i.e., 85.60%).

## Discussion

The purpose of this study was to consider how and to what extent cumulative advantages from SES shapes college attendance patterns. Analytically, this led us to focus on the extent to which family SES within a cohort of 10^th^ graders predicted the likelihood of attending college and attending a more selective college. As part of this, we examined primary pathways through which family SES may operate. Most notably, and unusually, we investigated relationships involving sport and non-sport extracurricular activity participation, as well as links to school contexts, college expectations, and test scores. We found evidence that family SES was a substantial and consistent predictor of the likelihood of attending college and attending a more selective college. Also, we found evidence that supported our expectations that college attendance patterns would be shaped by each of sport and non-sport extracurricular activities school contexts, college expectations, and test scores patterns—but that these mechanisms only appeared to explain a portion of the cumulative advantages for those from high family SES backgrounds.

First, we found consistent support for our initial hypothesis that family SES would be positively associated with college attendance and attendance at more selective colleges. Consistent with educational achievement research dating back to Coleman [33], family resources clearly play a major role in affecting student achievement. In fact, the relationships between family SES and college attendance patterns remained even after we accounted for school contexts, college expectations, students’ test scores, and participation levels in extracurricular activities. Overall, these findings support a cumulative advantage framework wherein family SES is recognized as shaping college attendance through its influence on school contexts, college expectations, academic abilities, and resume-building through extracurricular activities—but that there are still seemingly residual effects of family SES on college attendance patterns [26, 29, 31].

Our initial hypothesis regarding SES also emphasized the importance of SES-linked school contexts in predicting college attendance patterns. School level SES is correlated with having both additional resources and more high achieving peers, largely due to patterns of residential class segregation [5, 37]. As expected, we found evidence that school level SES is positively associated with college attendance and attendance at a more selective college. These findings support and extend previous research about the implications of family SES for high school contexts—but also offer new evidence of patterned links to college attendance, as well [26, 29, 31].

Our second hypothesis focused on assessing extracurricular activities as predictors of college attendance patterns. We found evidence that both sport and non-sport extracurricular participation independently increase the likelihood of attending college. At least in regards to sport participation, these benefits seem to accrue from both 10^th^ grade and 12^th^ grade participation. Yet, there is no evidence of an added benefit of participating in multiple sports or multiple non-sport activities. The potential for independent benefits from both sport and non-sport extracurricular activities has been suggested by previous scholars and we offer more advanced evidence of these dynamics in the present study [25, 66, 75–77]. In contrast to common assumptions that more extracurricular activities are better for youth development and resume building, we find that participating in multiple extracurricular activities does not increase the likelihood of attending college or a more select college. There is some support from other work for this conclusion, as well [27, 74].

Finally, our third hypothesis considered the relevance of college expectations and test scores. Indeed, we found that college expectations and test scores are positively associated with college attendance and attendance at a more selective college. The patterns of the results support previous evidence that these factors are extensions of family SES statuses and offer advantages for college attendance and attendance at more selective schools [9, 29, 31, 82]. Also, the inclusion of school context indicators, extracurricular involvement measures, college expectations, and academic ability measures altogether did not overwhelm family SES as a predictor of college attendance patterns, as anticipated.

Taken together, what do our results and hypotheses tell us about SES, college attendance, and social mobility? First and foremost is it important to state the obvious: family SES is important in predicting college attendance patterns. Inequalities by race/ethnicity and language largely evaporate once SES is taken into account. Also, this study offers novel evidence of how extracurricular activities may increase the likelihood of attending college and a more selective college, with both sports and non-sport activities providing advantages for attending any college as well as the likelihood of attending a more selective college. Higher SES parents are more likely to enroll their children in extracurricular activities, and they are more likely to live in contexts where these extracurricular activities are available [9, 12, 65]. Participating in extracurricular activities have consistently provided academic benefits over time [76], and a recent divergence in participation rates by socioeconomic status is alarming given how the patterns mirror inequalities in college attendance [11, 73].

In addition, our analysis suggests that structural factors such as school SES compositions impact students’ college attendance patterns. Family SES can determine the quality and type of school that students attend, which illustrates a cumulative advantage sequence. Still, growing up in a household with parents who attended college, and who hold high status professional jobs is likely to confer, whether explicitly or implicitly, the expectation that college is both attainable and expected. Parents with higher SES are also more likely to be able to nurture their children’s academic abilities. This may stem from their own knowledge, habits, interests, and even abilities to pay for classes, tutors, and other forms of assistance that can help their children to score higher on standardized tests, which increase the likelihood of college attendance and attendance at a more selective college. In sum, our analysis finds that all of these anticipated factors indeed correlate with college attendance patterns, as expected, with especially unique evidence from the present study for the significance of extracurricular activities. Yet, SES remains quite predictive of college attendance patterns, still. Subsequent research should continue to identify mechanisms advantaging those from high SES backgrounds. We hope these investigations will consider how individual factors are linked to structural conditions.

Nevertheless, our study does have limitations. These data are older than what would be ideal, and scholars should revisit these questions upon the arrival of subsequent nationally representative datasets. Also, an analysis leveraging the longitudinal nature of these data would be informative, and it would be preferable to have more insight into what transpires after the 10^th^ grade for the students in this study; however, there was very limited data for our variables of interest in subsequent waves. Additionally, because cumulative advantages begin well before the 10^th^ grade, it would be useful to have a measure of family SES at birth, for instance. Additional measures of the mechanisms through which SES is expected to operate would also be useful. These should include extensions of our emphases in this study, such as the availability of books while growing up and students’ participation in test prep classes; but, also, measures of stressors that disproportionately affect lower SES students such as exposures to violence, hunger, and poor health conditions. Furthermore, it may be that extracurricular activities are correlated with college attendance patterns, in part, through selection effects (e.g., harder workers and more competent students may be more likely to participate and become more likely to attend more selective colleges); future research might seek to better address this concern with more sophisticated analytical means of addressing this possibility. Still, such participation may importantly signal such traits to college admission officers [26]. Finally, in-depth interviews and ethnographic approaches could offer much needed complements to our approach and could help to further elucidate what motivates parents and children from different backgrounds; what pursuits occur, and why, amidst different opportunities, barriers, and challenges; and how educational and extracurricular systems and personnel respond to and support people from different backgrounds and with different experiences and achievements [9, 26, 65].

Nevertheless, this study is able to offer new and compelling information about the (dis)advantages that stem from family SES. College degrees have been one of the few credentials that allow individuals from lower class backgrounds to compete economically with those who grew up in more socioeconomically advantaged families. Additionally, returns to college vary by the selectivity of the institution [30]. Thus, providing access to college, and establishing more socioeconomic parity in attending selective colleges, is crucial to reducing inequalities in the U.S. Understanding the disparities and influences of family SES is central, in this regard.

### Implications

The findings of this study present both theoretical and practical implications. Overall, these findings support a cumulative advantage framework wherein family SES is recognized as shaping college attendance through its influence on school contexts, college expectations, academic abilities, and resume-building through extracurricular activities—but that there are still seemingly residual effects of family SES on college attendance patterns [26, 29, 31]. Our findings demonstrate that SES related experiences gained earlier in school careers yield increased chances for improved educational outcomes. Additionally, these results provide further evidence in support of Lareau’s recognized process of *concerted cultivation* and Friedman’s emphasis on parents working to nurture *competitive kid capital* [9, 65]. Higher SES families especially encourage their children to participate in a broad range of extracurricular activities in order to develop well-rounded individuals. The associations between extracurricular participation and attending college as well as more selective colleges provide supporting evidence for patterns previously identified in the literature. It is of interest, however, that there is a lack of evidence that participating in multiple sport or non-sport extracurricular activities helps students attend superior post-secondary schools. This suggests that overdoing extracurricular pursuits may not be particularly helpful, but there seems to be some benefit in becoming skilled, committed, and well-rounded with at least one sport and/or non-sport extracurricular activity.

Practically, these results highlight the necessity to provide equitable funding and resources in U.S. primary and secondary schools—for both educational and extracurricular environments and pursuits. Also, an intentionality to offset SES advantages in the pursuit of higher education. High SES families leverage the current system to cluster together in neighborhoods to inflate the resources of the schools their children attend. This practice leads to better teachers, more learning-oriented environments, higher expectations, and more resources for development as part of the education curriculum and outside of the classroom, in the form of extracurricular activities. They also are able to employ their own resources to produce hallmarks of preparedness and excellence in their offspring [49, 83]. Despite practical and legal barriers, and political resistance, to increase equity in educational funding and allow for more equity in the pursuit of higher education, addressing imbalances in funding and resources is crucial to reducing inequality in the United States. A more comprehensive approach may entail addressing socioeconomic inequalities in society, more generally.
